# The freezing Rènyi quantum discord

**DOI:** 10.1038/s41598-019-51206-9

**Published:** 2019-10-14

**Authors:** Xiao-Yu Li, Qin-Sheng Zhu, Ming-Zheng Zhu, Hao Wu, Shao-Yi Wu, Min-Chuan Zhu

**Affiliations:** 1 School of information and software engineering, University of Electronic Science and Technology of China, Chengdu, 610054 P. R. China; 2 School of Physics, University of Electronic Science and Technology of China, Chengdu, 610054 P. R. China

**Keywords:** Quantum information, Theoretical physics

## Abstract

As a universal quantum character of quantum correlation, the freezing phenomenon is researched by geometry and quantum discord methods, respectively. In this paper, the properties of Rènyi discord is studied for two independent Dimer System coupled to two correlated Fermi-spin environments under the non-Markovian condition. We further demonstrate that the freezing behaviors still exist for Rènyi discord and study the effects of different parameters on this behaviors.

## Introduction

As an important part of the quantum theory, the quantum correlation has aroused extensive attention in lots of physical fields, such as quantum information^1–3^, condensed matter physics^4,5^ and gravitation wave^6^ due to some unimaginable properties in a composite quantum system which can not be reproduced by a classical system. In the past twenty years, entanglement was considered as the quantum correlation and gradually understood. But, the quantum discord concept has been put forward by Ollivier and Zurek^7,8^ and Henderson and Vedral^9^ with the deep understanding of quantum correlation. It was clearly demonstrated that entanglement represents only a portion of the quantum correlations and can entirely cover the latter only for a global pure state^10^. Later, many efforts have been devoted to quantify quantum correlation from the view of geometry^10–20^ and entropy^7–9,21–29^.

Since the systematic correlation contains two parts: the classical correlations and quantum correlation, Maziero *et al*.^30^ found the frozen behavior of the classical correlations for phase-flip, bit-flip, and bit-phase flip channels. As for the possible similar behaviors for the quantum correlation, Mazzola, Piilo, and Maniscalco^31^ displayed the similar behavior of the quantum correlations under the nondissipative-independent-Markovian reservoirs for special choices of the initial state. In the same year, Lang and Caves^32^ provided a complete geometry picture of the freezing discord phenomenon for Bell-diagonal states. Later, some effort has been devoted to discuss the condition for the frozen-discord with some Non-Markovian processes and inial states^10,19,20,33^ (Bell-diagonal states, X states and SCI atates). In conclusion, the freezing discord shows a robust feature of a family of two-qubit models subject to nondissipative decoherence, that is, the quantum correlation does not change for a while. Although different measures of discords lead to some different conditions for the freezing phenomenon, seeing NMR experiment where the freezing discord was demonstrated^34^, this phenomenon of quantum correlation reflects a deeper physical interpretation, such as some relationship with quantum phase transition^35^. Recently, the Rènyi entropy^36,37^

1 $${S}_{\alpha }(\rho )=\frac{1}{1-\alpha }\,\log \,Tr[{\rho }^{\alpha }]$$

arouses much attention in the quantum information field. This comes from two aspects: (1) Certain properties of a quantum state $\rho$ which associated to density matrix elements can be quantified in terms of linear or nonlinear functions of $\rho$, such as the average values $TrA\rho$ of observable $\{A\}$ (linear functionals), the von Neumann entropy and the Rènyi entropy (nonlinear functions). Simultaneously, quantum networks can be used to estimate nonlinear functions of $\rho$ more directly and bypass tomography^38^. (2) The Rènyi entropy shows quantitative bounds for different parameter *α* comparing the von Neumann entropy, and it is easier to implement than the von Neumann entropy for measuring entanglement^39–42^. Here the parameter $\alpha \in (0,1)\cup (1,\infty )$ and the logarithm is in base 2. Notably, the R*é*nyi entropy will reduce to the von Neumann entropy when $\alpha \to 1$. As an natural extension of quantum discord, the Rènyi entropy discord (RED)^36,37,43–45^ is also put forward. Therefore, it is valuable to study the properties of RED and the condition for the freezing phenomenon of RED in quantum information field.

## The Quantum Correlation of Dimer System

### The definition of Rènyi discord

At first, Ollivier and Zurek^7,8^ gave the concept of quantum discord (QD)

2 $$D({\rho }_{AB})=\mathop{{\min }}\limits_{{\Pi }_{k}^{A}}\,\sum _{k}\,{p}_{k}S({\rho }_{k}^{B})+S({\rho }_{B})-S({\rho }_{AB})$$

to quantify the quantum correlation, where the von Neumann entropy $S(X)=-\,tr({\rho }_{X}\,{\log }_{2}\,{\rho }_{X})$ is for the density operator ${\rho }_{X}$ of system *X*, ${\rho }_{A(B)}=T{r}_{B(A)}({\rho }_{AB})$ is the reduced density matrix by tracing out the degree of the system *B*(*A*), ${p}_{k}=Tr({({\Pi }_{k}^{A})}^{\dagger }{\rho }_{AB}{\Pi }_{k}^{A})$ and ${\rho }_{k}^{B}=T{r}_{A}({({\Pi }_{k}^{A})}^{\dagger }{\rho }_{AB}({\Pi }_{k}^{A})/{p}_{k}$. Here, ${\Pi }_{k}^{A}$ denotes the measurement of system A.

Later, an equivalent description is introduced in refs ^36,37,43,44^. The main idea of this equivalent description is to apply an isometry extension of the measurement map ${U}_{A\to EX}$ from *A* to a composite system *EX*. This method reveals that any channel from *A* to *A*′ can be used to describe the composite system *EX* when we discard the freedom of *E*. Finally, the quantum discord is rewritten as:

3 $$D({\rho }_{AB})=\mathop{inf}\limits_{{\Pi }_{k}^{A}}I{(E;B|X)}_{{\tau }_{XEB}}$$

where the optimization is with respect to all possible POVMs ${\Pi }_{k}^{A}$ of system A with the classical output X. E is an environment for the measurement map and

4 $$\begin{array}{rcl}{\tau }_{XEB} & = & {U}_{A\to EX}{\rho }_{AB}{U}_{A\to EX}^{\dagger }\\ {U}_{A\to EX}|{\psi }_{A}\rangle  & = & \sum _{k}\,|k{\rangle }_{X}\otimes {(\sqrt{{\Pi }_{k}^{A}}|{\psi }_{A}\rangle \otimes |k\rangle )}_{E}\end{array}$$

The conditional mutual information $I{(E;B|X)}_{{\tau }_{XEB}}$ satisfy:

5 $$\begin{array}{rcl}I{(E;B|X)}_{{\tau }_{XBE}} & = & S{({\rho }_{EX})}_{{\tau }_{XEB}}+S{({\rho }_{BX})}_{{\tau }_{XEB}}\\  &  & -\,S{({\rho }_{X})}_{{\tau }_{XEB}}-S{({\rho }_{XEB})}_{{\tau }_{XEB}}\end{array}$$

where $S({\rho }_{EX})$denotes the von Neumann entropy of the composite system *EX* for total system EXB which has density matrix ${\tau }_{XEB}$. Similar definitions for $S({\rho }_{BX})$, $S({\rho }_{X})$ and $S({\rho }_{XEB})$.

As an extension of quantum discord, the Rènyi quantum discord of ${\rho }_{AB}$ is defined for $\alpha \in (0,1)\cup (1,2]$ as^43^

6 $${D}_{\alpha }({\rho }_{AB})=\mathop{inf}\limits_{{\Pi }_{k}^{A}}{I}_{\alpha }{(E;B|X)}_{{\tau }_{XEB}}$$

where the Rènyi conditional mutual information ${I}_{\alpha }{(E;B|X)}_{{\tau }_{XEB}}$ satisfy:

7 $$\begin{array}{rcl}{I}_{\alpha }{(E;B|X)}_{{\tau }_{XBE}} & = & \frac{\alpha }{\alpha -1}\,\log \,Tr\{({\rho }_{X}^{\frac{\alpha -1}{2}}T{r}_{E}\{{\rho }_{EX}^{\frac{1-\alpha }{2}}{\rho }_{EBX}^{\alpha }\\  &  & {{\rho }_{EX}^{\frac{1-\alpha }{2}}\}{\rho }_{X}^{\frac{\alpha -1}{2}})}^{\frac{1}{\alpha }}\}\end{array}$$

In this paper, we choose the von Neumann measurement ${\Pi }_{i^{\prime} }=|i^{\prime} \rangle \langle i^{\prime} |(i=0,1)$ with two angular parameters *θ* and $\varphi$: $|0^{\prime} \rangle =\,\cos (\theta /2)|0\rangle +{e}^{i\varphi }\,\sin (\theta /2)|1\rangle$ and $|1^{\prime} \rangle =\,\sin (\theta /2)|0\rangle -{e}^{i\varphi }\,\cos (\theta /2)|1\rangle$ ($0\le \theta \le \pi /2;0\le \varphi \le \pi$). The properties of the Rènyi quantum discord are shown in Table 2 of ref.^43^.

### The Hamiltonian of the open system

**Figure 1:**
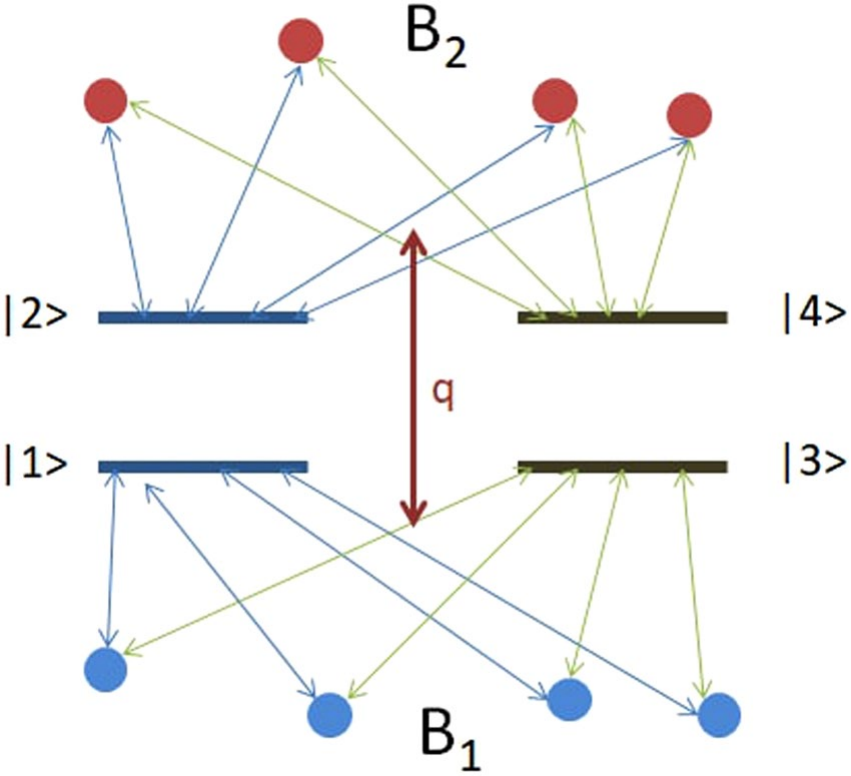
The two dimer systems interacting with two interaction spin-environments (*S*_1_ and *S*_2_). The red and blue balls denote the spin particles of environments (*B*_1_, *B*_2_), respectively. The $\mathrm{|1}\rangle$ and $\mathrm{|2}\rangle$ ($\mathrm{|3}\rangle$ and $\mathrm{|4}\rangle$) denote the energy level states of dimer system ${H}_{{d}_{1}}$ (${H}_{{d}_{2}}$). *q* denotes the interaction strength between the spin-environments *S*_1_ and *S*_2_. The arrows between the energy level states and spin particles of environments denote the interaction between dimer systems and environments.

We consider two independent dimer systems which are coupled to two correlated Fermi-spin environments, respectively, as shown in Fig. 1. The Hamiltonian of the total system has the following form^46^:

8 $$H={H}_{d}+\sum _{i=1,2}\,{H}_{{B}_{i}}+\sum _{i,j=1,2}\,{H}_{{d}_{i}{B}_{j}}+q{S}_{1}^{z}{S}_{2}^{z}$$

where ${H}_{d}={H}_{{d}_{1}}+{H}_{{d}_{2}}$ and ${H}_{{B}_{i}}$ describe two independent dimer system and Fermi-spin environments, respectively. ${H}_{{d}_{i}{B}_{j}}$ represent the interaction between the dimer and the spin environment; while ${S}_{1}^{z}{S}_{2}^{z}$ denotes the interaction between two spin environments. The collective spin operators are defined as ${S}_{i}^{z}={\sum }_{k=1}^{{N}_{i}}\,\frac{{\sigma }_{z}^{k,i}}{2}$, where ${\sigma }_{z}^{k,i}$ are the Pauli matrices and *α*_*i*_ is the frequency of ${\sigma }_{z}^{k,i}$. So $q{S}_{1}^{z}{S}_{2}^{z}$ describes an Ising-type correlation between the environments with strength *q*. The cases $q=0$ and $q\ne 0$, describe independent and correlated spin bath, respectively. The various parts of the Hamiltonian can be written as following forms:

$$\begin{array}{rcl}{H}_{{d}_{1}} & = & {\varepsilon }_{1}|1\rangle \langle 1|+{\varepsilon }_{2}|2\rangle \langle 2|+{J}_{1}(|1\rangle \langle 2|+|2\rangle \langle 1|)\\ {H}_{{d}_{2}} & = & {\varepsilon }_{3}|3\rangle \langle 3|+{\varepsilon }_{4}|4\rangle \langle 4|+{J}_{2}(|3\rangle \langle 4|+|4\rangle \langle 3|)\\ {H}_{{d}_{1}{B}_{1}} & = & {\gamma }_{1}|1\rangle \langle 1|{S}_{1}^{z};{H}_{{d}_{1}{B}_{2}}={\gamma }_{2}|2\rangle \langle 2|{S}_{2}^{z}\\ {H}_{{d}_{2}{B}_{1}} & = & {\gamma }_{3}|3\rangle \langle 3|{S}_{1}^{z};{H}_{{d}_{2}{B}_{2}}={\gamma }_{4}|4\rangle \langle 4|{S}_{2}^{z}\\ {H}_{{B}_{i}} & = & {\alpha }_{i}{S}_{i}^{z}\end{array}$$

Here, each environment *B*_*i*_ consists of *N*_*i*_ particles $(i=1,2)$ with spin $\tfrac{1}{2}$; ${\varepsilon }_{a}$ and $|a\rangle$ ($a=1,2,3,4$) are the energy levels and the energy states of the dimer system, *J*_1_ and *J*_2_ are the amplitudes of transition. The interaction intensity between the spin particle and the environment is *γ*_*i*_

### The dynamics evolution of the dimer system

The formal solution of the von Neumann equation ($\hslash =1$)

9 $$\frac{d}{dt}\rho (t)= {\mathcal L} \rho (t)=-\,i[H,\rho (t)]$$

can be solved as

10 $$\rho (t)={e}^{ {\mathcal L} t}\rho (0)$$

where $\rho (t)$ denotes the density matrix of the total system.

The dynamics of the reduced density matrix ${\rho }_{d}(t)$ is obtained by the partial trace method which discards the freedom of the environments. That is

11 $${\rho }_{d}(t)=T{r}_{B}({e}^{ {\mathcal L} t}\rho (0))$$

Here, the states $|j,m\rangle$ denote the orthogonal bases in the environment Hilbert space *H*_*B*_ which satisfy^47^:

$$\begin{array}{rcl}{S}^{2}|j,m\rangle  & = & j(j+1)|j,m\rangle ;\\ {S}^{z}|j,m\rangle  & = & m|j,m\rangle ;{S}^{2}={({S}^{x})}^{2}+{({S}^{y})}^{2}+{({S}^{z})}^{2}\\ j & = & 0,\ldots ,\frac{N}{2};m=j,\ldots ,-\,j\end{array}$$

For the initial state $\rho (0)={\rho }_{d}(0)\otimes {\rho }_{B}(0)$ condition, the reduced density matrices ${\rho }_{d}(t)$ of the dimer system is

12 $$\begin{array}{rcl}{\rho }_{d}(t) & = & \frac{1}{Z}\,\mathop{\sum }\limits_{{j}_{1}=0}^{{N}_{1}/2}\,\mathop{\sum }\limits_{{m}_{1}=-{j}_{1}}^{{j}_{1}}\,\mathop{\sum }\limits_{{j}_{2}=0}^{{N}_{2}/2}\,\mathop{\sum }\limits_{{m}_{2}=-{j}_{2}}^{{j}_{2}}\,\frac{\nu ({N}_{1},{j}_{1})\nu ({N}_{2},{j}_{2})}{{e}^{\beta q{m}_{1}{m}_{2}}{e}^{\beta {\alpha }_{1}{m}_{1}}{e}^{\beta {\alpha }_{2}{m}_{2}}}\\  &  & \times \,{A}^{\dagger }{U}^{\dagger }{\rho ^{\prime} }_{d}(0)UA\end{array}$$

where $\nu ({N}_{i},{j}_{i})$ denotes the degeneracy of the spin bath^47–49^. ${\rho ^{\prime} }_{d}(0)$ is the matrix form of the density operator ${\rho }_{d}(0)$ under the basis states of dimer system Hilbert space ${A}^{\dagger }=(\langle 3|\langle 1|\langle 3|\langle 2|\langle 4|\langle 1|\langle 4|\langle 2|)$. The symbol *U* in Eq. (12) denotes the 4 × 4 matrix and equal to *MBQ* (here, *M*, *B* and *Q* are also 4 × 4 matrices^46^).

In order to obtain Eq. (12), the environment is given as the canonical distribution

$${\rho }_{B}(0)=\frac{1}{Z}{e}^{\beta q{S}_{1}^{z}{S}_{2}^{z}}\,\mathop{\prod }\limits_{i=1}^{2}\,{e}^{-\beta {\alpha }_{i}{S}_{i}^{z}}$$

with $\beta =\tfrac{1}{{K}_{B}T}$ (*T* is temperature and *K*_*B*_ is Boltzmann constant). The partition function *Z* is

$$Z=\mathop{\sum }\limits_{{j}_{1}=0}^{{N}_{1}/2}\,\mathop{\sum }\limits_{{m}_{1}=-{j}_{1}}^{{j}_{1}}\,\mathop{\sum }\limits_{{j}_{2}=0}^{{N}_{2}/2}\,\mathop{\sum }\limits_{{m}_{2}=-{j}_{2}}^{{j}_{2}}\,\frac{\nu ({N}_{1},{j}_{1})\nu ({N}_{2},{j}_{2})}{{e}^{\beta q{m}_{1}{m}_{2}}{e}^{\beta {\alpha }_{1}{m}_{1}}{e}^{\beta {\alpha }_{2}{m}_{2}}}$$

### The properties of Rènyi discord

In this section, the changing behaviors of the quantum correlation are discussed for the two-qubit X^10^ and special canonical initial (SCI)^33^ under different parameters, respectively. The two-qubit X state is widely used in condensed matter systems and quantum dynamics^10–12,22,28,29,50–54^. Under the basis vectors $|00\rangle$, $|01\rangle$, $|10\rangle$ and $|11\rangle$ (here 0(1) denotes the spin up (down) state), the density matrix of a two-qubit X state can be written as

13 $${\rho }_{d}(0)=[\begin{array}{cccc}a & 0 & 0 & \delta \\ 0 & b & \beta  & 0\\ 0 & {\beta }^{\ast } & c & 0\\ {\delta }^{\ast } & 0 & 0 & d\end{array}]$$

satisfying $$a,b,c,d\ge 0$$, $$a+b+c+d=1$$, $$\parallel \delta {\parallel }^{2}\le ad$$ and $$\parallel \beta {\parallel }^{2}\le bc$$.

Unlike X states, the class of canonical initial (CI)states^33^ have the density matrix

14 $${\rho }_{d}(0)=\frac{1}{4}[\begin{array}{cccc}1+{C}_{33} & {C}_{01} & {C}_{10} & {C}_{11}-{C}_{22}\\ {C}_{01}^{\ast } & 1-{C}_{33} & {C}_{11}+{C}_{22} & {C}_{10}\\ {C}_{10}^{\ast } & {C}_{11}+{C}_{22} & 1-{C}_{33} & {C}_{01}\\ {C}_{11}-{C}_{22} & {C}_{10}^{\ast } & {C}_{01}^{\ast } & 1+{C}_{33}\end{array}]$$

and the SCI states satisfy:

15 $$\{\begin{array}{rcl}{C}_{22}/{C}_{33} & = & -\,{C}_{11}\\ {C}_{10}/{C}_{01} & = & {C}_{11}\\ {({C}_{33})}^{2}+{({C}_{01})}^{2} & \le  & 1\end{array}$$

In view of the freezing phenomenon for X (SCI) states^10–12,19,22,28,29,33,50–54^ by geometry and von Neumann entropy discords, X and SCI initial states are chosen here.

**Figure 2:**
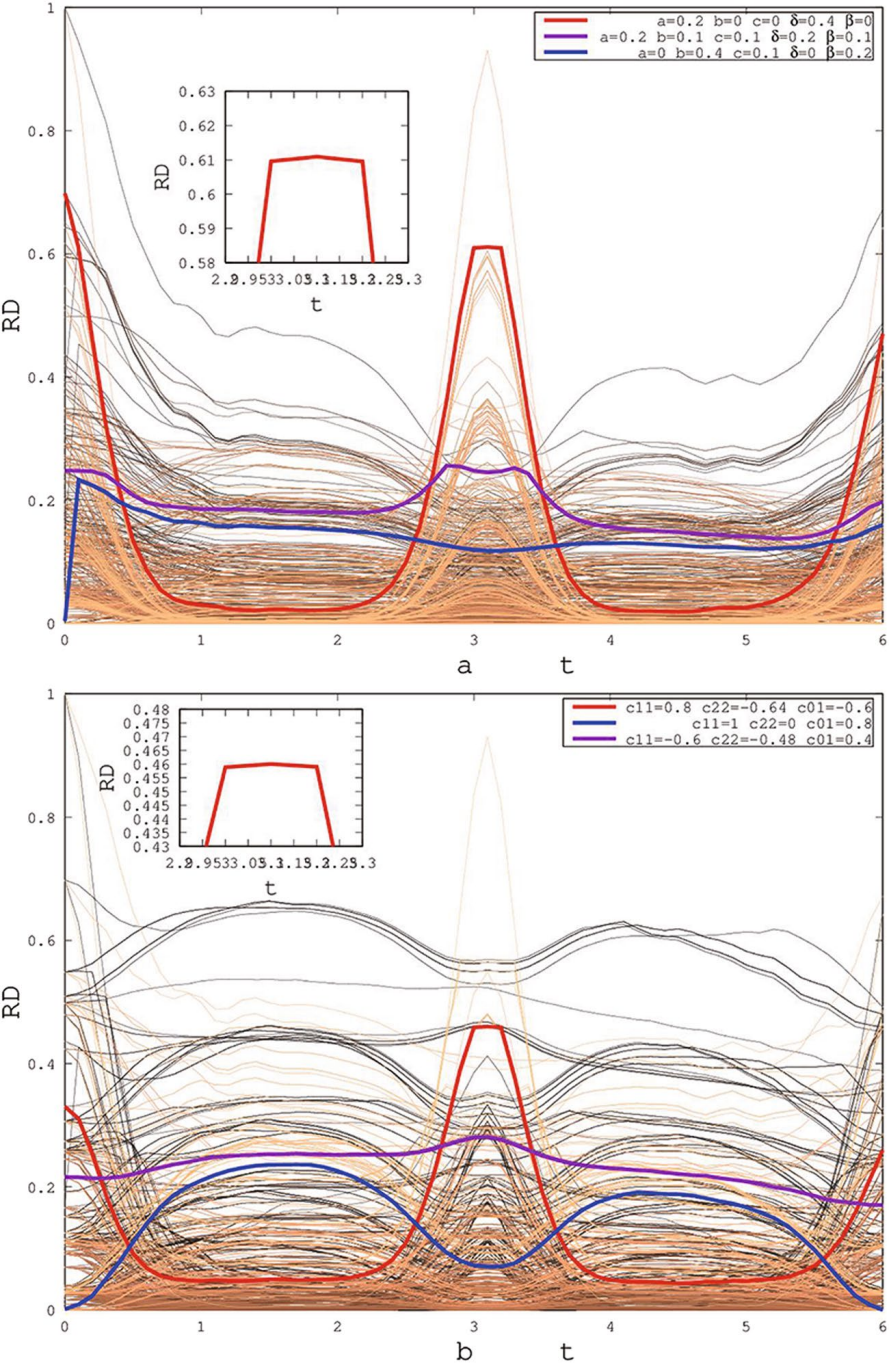
The properties of Rènyi discord as a function of time *t* for X and SCI initial states. The parameters are ${\alpha }_{1}=250\,p{s}^{-1}$, ${\alpha }_{2}=200\,p{s}^{-1}$, ${\Delta }_{1}=20\,p{s}^{-1}$, ${\Delta }_{2}=10\,p{s}^{-1}$, ${\Delta }_{3}=22\,p{s}^{-1}$, ${\Delta }_{4}=12\,p{s}^{-1}$, $q=30\,p{s}^{-1}$, $\beta =1/77$, ${N}_{1}=20$, ${N}_{2}=22$, ${\gamma }_{1}=1\,p{s}^{-1}$, ${\gamma }_{2}=1.1\,p{s}^{-1}$, ${\gamma }_{3}=0.9\,p{s}^{-1}$, ${\gamma }_{4}=1.2\,p{s}^{-1}$, ${J}_{1}=10\,p{s}^{-1}$, ${J}_{2}=12\,p{s}^{-1}$ and $\alpha =0.9$.

In Fig. 2, the changing behaviors of quantum correlation are shown for X (Fig. 2(a)) and SCI (Fig. 2(b)) initial states. Every line describes the changing behaviors of quantum correlation for one initial states. With the time evolution, the quantum correlation displays the non-Markov behaviors, especially the peak at about 3 second for some initial states, e.g. red line. It indicates the feedback of quantum information (quantum correlation) with the non-Markov process. When the quantum correlation value fluctuates within the range of 10^−3^ for numerical computation of a computer, we believe that the freezing of quantum correlation occurs. Because there exists the freezing of quantum correlation for some initial states, it is shown quantum correlation of two initial states which exist the freezing phenomenon in Fig. 2. The purple line shows longer freezing phenomenon contrasting with the red line. The red line has larger value of quantum correlation for the freezing platform. It also hints that the freezing phenomenon of quantum correlation is a universal quantum character and has a deep physical meaning. In the inset box, it shows the partially enlarged drawing of the max freezing quantum correlation. Simultaneously, the blue solid line shows the spring of the quantum correlation for the initial states with zero quantum correlation at *t* = 0. It means that we can generate the quantum correlation by the environments of this quantum system. From the physical perspective of information flow, the total correlation of the system changes as the system information flows between the system and the environment, for example, the system information is always lost under the Markov process, and there is the feedback behavior under the non-Markov process. Since the total correlation consists of classical correlation and quantum correlation, in the flow of information, if only the classical correlation changes, then the quantum correlation exists freezing phenomenon.

**Figure 3:**
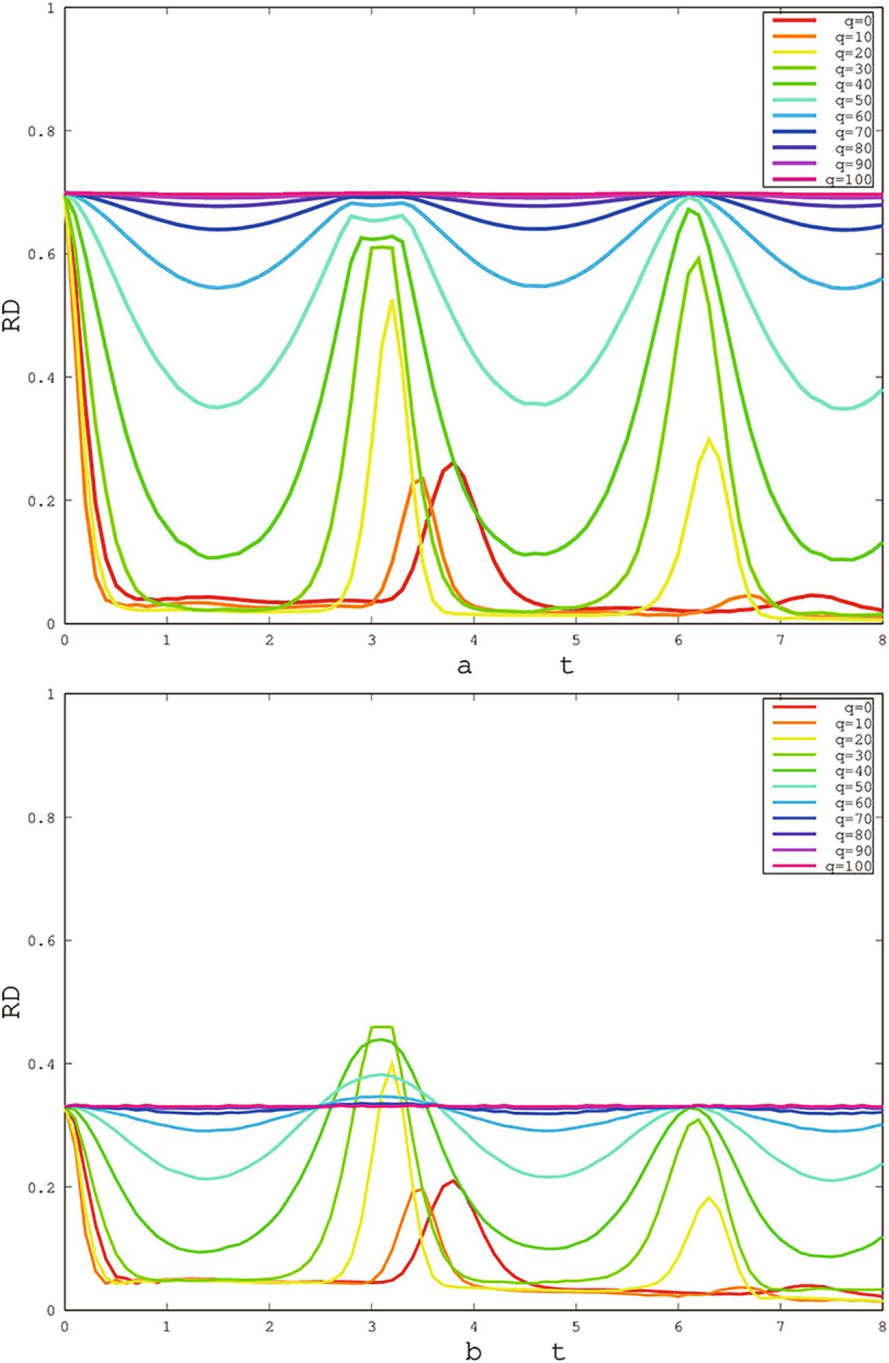
The changes of freezing Rènyi discord based on X (**a**) and SCI (**b**) initial states (Here, choosing the initial states of red solid line of Fig. 2) with time *t* for different environment coupling parameters *q*. The other parameters are same in Fig. 2.

Although the general results are obtained, the intrinsic parameters may play an important role in the changing behaviors of the quantum correlation^19,20,21,55^, especially the freezing behavior. In Fig. 3, the environment coupling parameter *q* can strongly affect the occurrence of freezing phenomenon, and the quantum correlation appears the quasi-periodic oscillations for X (Fig. 3(a)) and SCI (Fig. 3(b)) initial states. However, the oscillation behaviors are depressed with *q* ≥ 50 for X and SCI initial states. The freezing phenomena of quantum correlation also spring up with increasing *q* for X state. But, the freezing phenomena of SCI state first appears with increasing *q*, and then disappears with *q* ≥ 70. Particularly, the freezing phenomena always exist throughout the time when *q* excesses 90. Furthermore, the value of quantum correlation increases with increasing *q* for X state. From the perspective of the non-Markovian dynamical process, the larger *q* means the more information flowing from the system into the environment than that from the environment into the system. Therefore, a reasonable value of *q* is important for the maintenance of quantum correlation.

**Figure 4:**
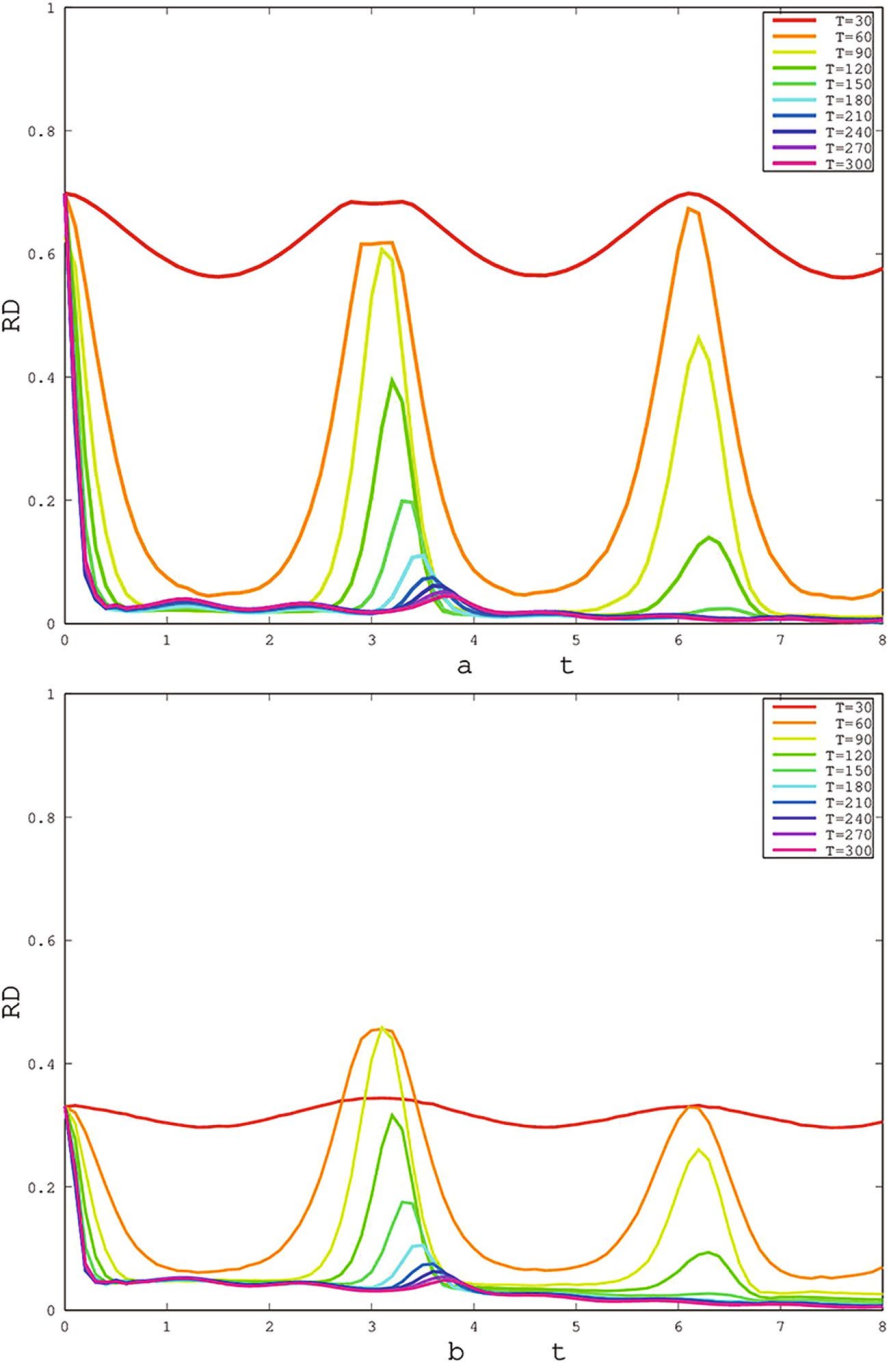
The changes of freezing Rènyi discord based on X (**a**) and SCI (**b**) initial states (Here, choosing the initial states of red solid line of Fig. 2) with time *t* for different environment temperatures *T*. The other parameters are same in Fig. 2.

Except for the parameter *q*, the temperature T is also important for the quantum correlation. According to our previous works^35–37^, a higher temperature may depress the activity of quantum correlation. How does temperature affect the frozen platform? The effect of temperature on the frozen platform is shown in Fig. 4. With increasing temperature *T*, the frozen platform collapses and then reappears at *T* ≥ 150. Simultaneously, the platform height is reduced.

**Figure 5:**
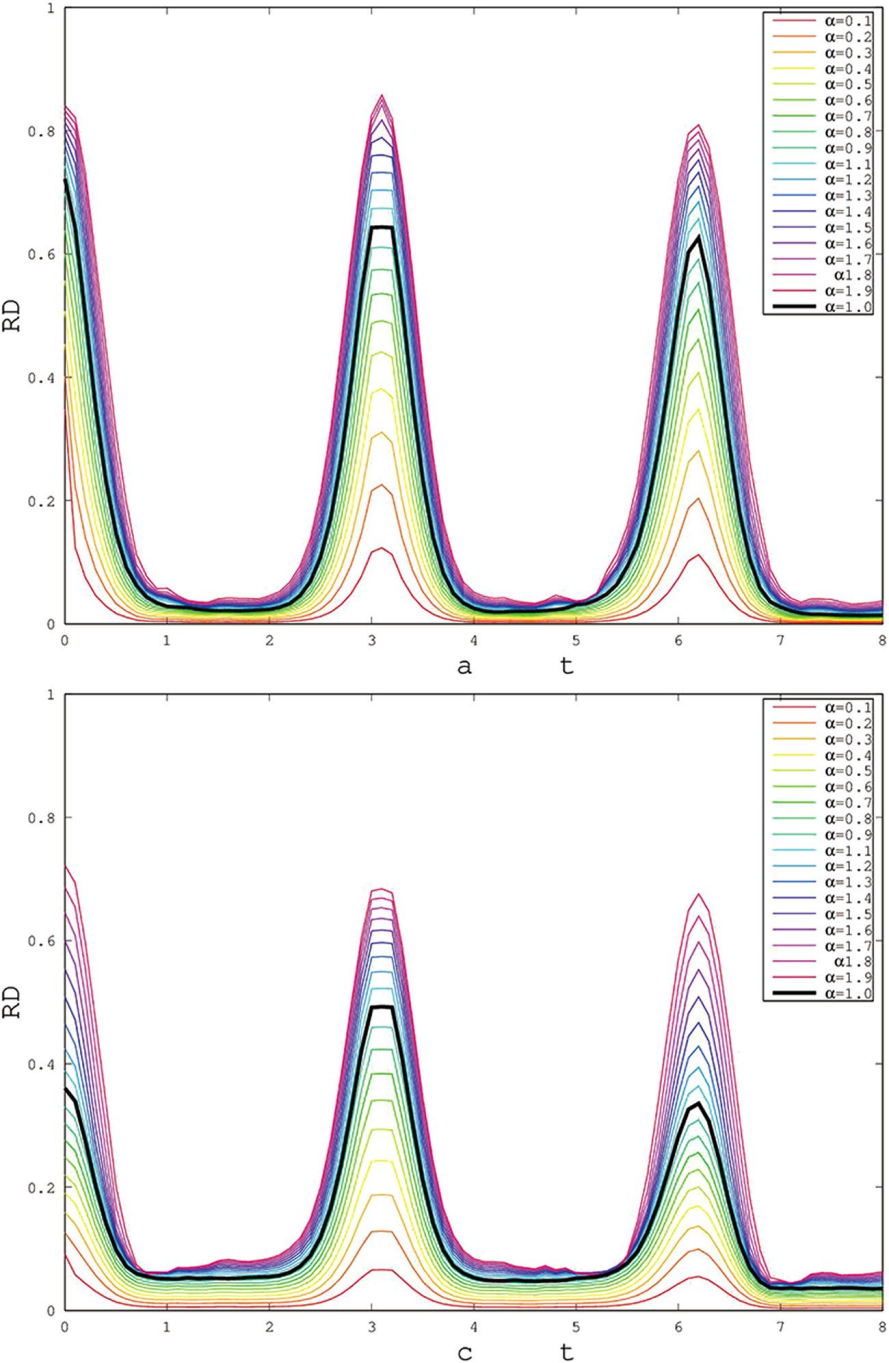
The changes of freezing Rènyi discord based on X (Fig. 4(a)) and SCI (Fig. 4(b)) initial states (Here, choosing the initial states of red solid line of Fig. 2) with time *t* for different parameter *α*. The other parameters are the same as Fig. 2.

Figure 5 display the effect of different parameters *α* which is an important parameter of Rènyi entropy for Rènyi discord. With the increase of *α*, the monotonicity of Rènyi discord is well displayed^36,37^. For X and SCI initial states, the freezing platform appears in the range of $\alpha \in [0.7,1.4]$ and $\alpha \in [0.1,1.6]$, respectively. When the freezing platform appears, there is a particular scope of parameter alpha, which depends on the initial states. It stem from: when we calculate the quantum correlation, the classical correlation is related to the quantification (measurement) method, so the freezing phenomenon is related to the measurement method. Finally, compared the black line ($\alpha =1$) with others, the quantum discord only shows part of the nature of quantum correlation which quantifies by one of entropy discord, while the others correspond to different entropy discord. This otherness maybe supply the help to discuss the difference between quantum discord and geometric discord, especially, the occurrence of freezing phenomenon has different conditions.

## Conclusion

In this paper, the Rènyi discord has been applied to study the properties of quantum correlation of two independent Dimer System which coupled to two correlated Fermi-spin environments for the first time. We also study the freezing behaviors of Rènyi discord for the first time. It show bona fide discord quantification methods all freeze under the certain condition for non-dissipative dynamics and depend on the quantification method (different *α*) and system parameters, e.g. coupling parameters *q* and temperatures *T*. Comparing the quantum discord, the Rènyi discord is more favorable to discuss the upper limit value of the freezing phenomenon and let us understand the robustness of the system.
